# Knowledge and practices regarding antibiotics use: Findings from a cross-sectional survey among Italian adults

**DOI:** 10.1093/emph/eoaa028

**Published:** 2020-07-30

**Authors:** Aida Bianco, Francesca Licata, Rossella Zucco, Rosa Papadopoli, Maria Pavia

**Affiliations:** e1 Department of Health Sciences, School of Medicine, University of Catanzaro Magna Græcia, Catanzaro 88100, Italy; e2 Department of Experimental Medicine, University of Campania Luigi Vanvitelli, Naples 80138, Italy

**Keywords:** antimicrobial resistance, antibiotic use, Italy, public, self-medication

## Abstract

**Background and objectives:**

This study aimed to assess the knowledge on antibiotics and antimicrobial resistance (AMR) and the antibiotic use among the general public in Southern Italy and to analyze whether sociodemographic characteristics could be associated with poor knowledge and improper practices.

**Methodology:**

From March to November 2019, a face-to-face interview was conducted with adult subjects attending the waiting room of 27 randomly selected general practitioners (GPs) in Southern Italy. The questionnaire covered sociodemographic characteristics, knowledge on antibiotics and AMR and practices regarding the consumption of and self-medication with antibiotics.

**Results:**

The response rate was 89.7%. In the sample, 29.2% thought that antibiotics are effective for viral infections, and 49.5% correctly recognized the definition of AMR. Predictors of good knowledge about antibiotics and AMR were female gender and a higher education level. Almost half of the respondents had used antibiotics in the previous year and 23.6% took antibiotics to treat a common cold and/or fever. Among participants, 25.5% reported to have bought antibiotics without a prescription, and 30.6% were classified as antibiotic self-medication users. Use of antibiotics in the previous 12 months and having taken an antibiotic after a phone consultation with the GP were positively associated with both antibiotic use for a common cold and/or fever and self-medication with antibiotics.

**Conclusions and implications:**

The findings of this study highlighted a considerable antibiotic consumption in the adult population of Southern Italy together with misconceptions regarding the correct indication for antibiotic use that could foster indiscriminate antibiotic use.

**Lay Summary:**

The findings of this study highlighted a considerable antibiotic consumption in the adult Italian population together with misconceptions regarding the correct indication for antibiotic use that could foster indiscriminate antibiotic use. Almost a quarter of the respondents took antibiotics to treat a common cold and/or fever and reported to have bought antibiotics without a prescription.

## BACKGROUND AND OBJECTIVES

Since their discovery, antibacterial drugs have become an essential part of the modern healthcare landscape, allowing treatment of previously life-threatening bacterial infections. However, the enormous and irresponsible use of the antibiotics in human, animal and environmental sectors has significantly contributed to the advent of resistant strains. The rapid emergence of antimicrobial resistance (AMR) is currently a worldwide public health crisis, and it has been cited by the World Health Organization as one of the top global public health problems [1, 2]. The cost of AMR is immense for human health and lives as well as for the economic impact. The Organization for Economic Co-operation and Development predicts that 2.4 million people in Europe, North America and Australia will die from infections due to resistant microorganisms in the next 30 years and could cost up to US$3.5 billion per year [3]. It also highlighted the need for countries to urgently put in place policies to tackle AMR and prevent its disastrous consequences.

Preserving the continued effectiveness of existing antimicrobials includes a combination of interventions, such as strengthening health systems and surveillance, improving hygiene and infection control and appropriate use of antimicrobials in hospitals and in the community [4–7]. Specific guidelines for AMR stewardship should be informed by patterns of antimicrobials prescribing by healthcare providers and by the general public’s antimicrobial consumption habits. Further evidence of public awareness about AMR and antibiotic consumption in the community is required to contextualize and optimize the effectiveness of interventions. Self-medication plays a major role in ever-increasing antibiotic consumption [8], and adherence to antibiotic treatment is not only important to ensure their therapeutic effect but also to prevent the development of AMR.

Despite available data on public perceptions of AMR and practices regarding antibiotic use in Southern Italy on subgroups of population (i.e. people who had taken antibiotics in 12 months before the study [9] or parents [10]) and in other countries [11–14], generalization of the published results may be difficult because antibiotic use is influenced by several components, such as behavioral factors, access to and availability of antimicrobials and national policies [15]. Understanding the current public knowledge and practice about antibiotics use and identifying misconceptions could help to shape policies and campaigns addressing these topics in the local context. Therefore, in the present study, we investigated the knowledge and practices regarding antibiotics use and AMR among the general population. A further aim of the study was to analyze whether sociodemographic characteristics of the population could be associated with poor knowledge and improper practices.

## METHODOLOGY

### Study design

This cross-sectional study was conducted between March and November 2019 in the Calabria and Sicily Regions of Southern Italy. We randomly selected 17 general practitioners (GPs) and 10 community-based pediatrician (CBP) from a list of geographical units provided by the health districts as being responsible for coordinating and providing primary care. All consecutive subjects were recruited at randomly selected days from those attending GP and CBP clinics, with 20–30 individuals selected from each physician’s clinic. Two trained physicians, not involved in patient care, conducted a structured face-to-face interview with the subjects attending the waiting room of the selected physicians. Data collectors assured the participants of confidentiality and informed them of the purpose and methods of the investigation, and a signed informed consent form was obtained from all participants who agreed to participate in the study.

All the information was self-reported by the participants, no medical records or interviews by any GP or CBP were used as sources of data. No incentives were offered for participation.

### Sample size

Sample size was determined in order to warrant estimation of proportions with an expected margin of error of 5%, assuming an intended confidence interval level (CI) of 95%. We used the prevalence of knowledge of antibiotics and AMR and practices regarding antibiotics use and self-medication obtained from similar studies [10, 16]. Based on these assumptions, a sample of at least 500 adults from the general population was required.

### Questionnaire design

The questionnaire was developed based on the literature review of comparable studies [10, 13, 16–21]. It was pretested and validated by a pilot study on a sample of 40 subjects not included in the study. Findings from this phase of the study provided the research team with feedback that confirmed adequate comprehension of the questions.

The questionnaire covered a broad range of issues related to sociodemographic characteristics of the participants (gender, age, nationality, marital status, education level and employment status, having at least a son/daughter and his/her age), knowledge on antibiotics and AMR and practices regarding the consumption of antibiotics.

Each question elicited responses in a variety of formats: yes/no/do not know, closed-ended with multiple answers possible and open options.

To assess knowledge, the participants were asked to respond to 10 different statements (six true statements and four false): two statements were related to the identification of antibiotics (Amoxicillin is an antibiotic/Paracetamol is an antibiotic); four related to antibiotic use with or without an indication (Antibiotics are effective for bacterial infections/Antibiotics are effective for viral infections/Antibiotics are indicated to reduce any kind of pain and inflammation/You can stop taking the antibiotic when you start feeling better); two were about the side effects of antibiotics (Antibiotics can kill ‘good bacteria’ of the human ecosystem/Antibiotics can cause allergic reactions); and two were regarding the awareness of AMR (AMR is a phenomenon that takes place when a bacterium loses its sensitivity to an antibiotic/Infection prevention measures limit the development of AMR). An overall knowledge score was calculated by assigning one point for each correct response and summing the scores to each statement. The total score ranged from 0 to 10. The overall median knowledge score of the respondents was then estimated and a ≤50th percentile score was interpreted as poor knowledge, whereas a >50th percentile score as good knowledge.

The respondents’ antibiotic use and self-medication practices were measured with questions asking if they and/or their minor son/daughter (i.e. younger than 18 years old) had been prescribed antibiotics within the previous year; frequency of prescribing in the same period; having ever used an antibiotic without indication (Have you ever taken an antibiotic for common cold?/Have you ever taken an antibiotic for a fever?); whether they had adhered to the GP’s prescription; whether they had ever stored and used leftover antibiotics; whether they had ever purchased an antibiotic without a prescription of the GP and whether they had ever begun an antibiotic therapy after a phone consultation with the GP, without a clinical examination. The responders were classified as antibiotic self-medication users if they reported that they had ever bought an antibiotic without a prescription from the GP and/or had ever used leftover antibiotics.

Ethical approval was granted by the Regional Human Research Ethics Committee (ID No. 122/2019/04/18).

### Statistical analysis

Statistical analysis was performed using STATA software program, version 16 [22]. Data were summarized using frequencies and percentages for categorical data and mean and SDs for continuous data. Bivariate analyses (*t*-test and *χ*^2^ test) were conducted to examine the unadjusted relationships between the outcomes of interest and the independent variables. Furthermore, multivariate analyses were conducted using stepwise multivariate logistic regression modeling techniques. All significant variables identified in the bivariate analysis, and other variables considered as potential determinants of outcomes of interest were included in the analysis. A significance level of 5% was used for hypothesis testing. The following outcomes of interest were investigated: knowledge about antibiotics and AMR (Model 1), antibiotic use in the previous 12 months (Model 2), antibiotic use for common cold and/or fever (Model 3) and self-medication with antibiotics (Model 4). The following variables were included in all models: gender (male = 0; female = 1), age (four categories in years: 18–35 = 1, 36–50 = 2, 51–65 = 3 and >65 = 4), nationality (Italian = 0, other = 1), education level (secondary school or lower = 1, high school = 2, bachelor/university/doctoral degree = 3), employment status (unemployed = 0, employed = 1), marital status (others = 0, married = 1) and having at least one minor son/daughter who had used antibiotics in the previous 12 months (no = 0, yes = 1). In Models 3 and 4, the following variables were included: knowledge about antibiotics and AMR (poor = 0, good = 1), use of antibiotics in the previous 12 months (no = 0, yes = 1), having interrupted an antibiotic course (no = 0, yes = 1), having taken an antibiotic after a phone consultation with the GP (no = 0, yes = 1). In Model 3, the independent variables having purchased an antibiotic without a prescription (no = 0, yes = 1) and storing leftover antibiotics (no = 0, yes = 1) were also included. We applied a procedure to obtain the final models with *P* values of >0.2 and <0.4 for the inclusion and exclusion of the variables in the models, respectively. Adjusted odds ratio (OR) and 95% CI were calculated.

The data set was deposited in Mendeley Data repository (doi:10.17632/6rkz746j35.1).

## RESULTS

### Participant demographics

Of the original sample of 633 subjects, 568 agreed to participate for a response rate of 89.7%.

More than half were female (58.8%), and the mean age observed was 47.8 years (±16.7). The majority held high school qualification (47.5%), was employed (53.7%) and had at least one minor son/daughter (58.1%).

### Knowledge of antibiotics and AMR

The overall median knowledge score was 8 (interquartile range 6–9), and only 17.1% of all the respondents gave the correct answer to all 10 statements.

Table 1 presents the answers to the statements about knowledge regarding antibiotics and AMR. When assessing the knowledge about identification of antibiotics, 456 out of 568 participants (80.3%) correctly answered both questions about antibiotics. Furthermore, 29.2% of the sample wrongly thought that antibiotics are effective for viral infection. Regarding the knowledge about correct use and side effects of antibiotics, 17.3% reported that ‘antibiotics are indicated to reduce any kind of pain and inflammation’ and 13.2% asserted that it is possible to stop taking antibiotics when a subject starts feeling better. Participants were weakly knowledgeable about the correct statements on AMR. Mass media (television, internet and newspapers) were the main source of information about AMR (50.8%), followed by healthcare workers (HCW) and colleagues/friends/family members (21.3%).

**Table 1.** Knowledge related to antibiotics and AMR

Statements (568 respondents)	Yes/true		No/false	
	*N*	%	*N*	%
Amoxicillin is an antibiotic	**508**	**89.5**	60	10.5
Paracetamol is an antibiotic	73	12.9	**495**	**87.1**
Antibiotics are effective for bacterial infections	**518**	**91.2**	50	8.8
Antibiotics are effective for viral infections	166	29.2	**402**	**70.8**
Antibiotics are indicated to reduce any kind of pain and inflammation	98	17.3	**470**	**82.7**
You can stop taking the antibiotic when you start feeling better	75	13.2	**493**	**86.8**
Antibiotics can kill ‘good bacteria’ of the human ecosystem	**370**	**65.2**	198	34.8
Antibiotics can cause allergic reactions	**510**	**89.8**	58	10.2
AMR is a phenomenon that takes place when a bacterium loses its sensitivity to an antibiotic	**281**	**49.5**	287	50.5
Infection prevention measures limit the development of AMR	**200**	**35.2**	368	64.8

Number and percentages referring to correct answers are in bold.

The model predicting good knowledge about antibiotics and AMR showed multiple significant independent sociodemographic associations. Every level of education from secondary school upward was positively associated with good knowledge. The effect size for high school (OR = 3.93, 95% CI = 2.45–6.29) was lower than bachelor/university/doctoral degree (OR = 10.09, 95% CI = 5.82–17.51). Female gender (OR = 1.76, 95% CI = 1.17–2.64) was positively associated and foreign nationality (OR = 0.25, 95% CI = 0.08–0.84) negatively associated with correct knowledge on antibiotics and AMR (Model 1 in Table 2). Having at least one minor son/daughter who had used antibiotics in the previous 12 months (OR = 1.51, 95% CI = 0.97–2.33) was not confidently presented as an independent indicator of good knowledge about antibiotics and AMR, although the low confidence bound proximates to an OR of 1 suggesting that an association cannot be entirely dismissed.

**Table 2.** Stepwise multivariate logistic regression models for potential determinants of the different outcomes of interest

Variables	OR	95% CI	*P*
*Model 1. Outcome: Good knowledge about antibiotics and AMR* ^a^			
*Log-likelihood = −338.61, χ^2^* = *107.66, P* < *0.0001, No. of obs. = 568*			
Education level			
Secondary school or lower^b^	1.00		
High school	3.93	2.45–6.29	<0.001
Bachelor/University/Doctoral degree	10.09	5.82–17.51	<0.001
Gender			
Male^b^	1.00		
Female	1.76	1.17–2.64	0.006
Nationality			
Italian^b^	1.00		
Other	0.25	0.08–0.84	0.024
Having at least one minor son/daughter who had used antibiotics in the previous 12 months			
No^b^	1.00		
Yes	1.51	0.97–2.33	0.066
Age, ordinal	1.18	1.17–2.64	0.105
Marital status			
Others^b^	1.00		
Married	0.76	0.46-1.23	0.260
*Model 2. Outcome: Antibiotic use in previous 12 months* ^c^			
*Log-likelihood = −383.65, χ* ^2^ *= 19.10, P* < *0.0001, No. of obs.* = *568*			
Having at least one minor son/daughter who had used antibiotics in the previous 12 months			
No^b^	1.00		
Yes	1.74	1.19–2.54	0.004
Employment status			
Unemployed^b^	1.00		
Employed	0.65	0.46–0.92	0.014
Education level			
Secondary school or lower^b^	1.00		
High school	1.34	0.95–1.88	0.093
Bachelor/University/Doctoral degree	Backward elimination		
Marital status			
Others^b^	1.00		
Married	0.75	0.47–1.17	0.206
*Model 3. Outcome: Antibiotic use for common cold and/or fever* ^d^			
*Log-likelihood = −278.56, χ* ^2^ *= 63.50, P* < *0.0001, No. of obs.* = *568*			
Having taken an antibiotic after a phone consultation with the GP			
No^b^	1.00		
Yes	2.16	1.41–3.33	<0.001
Use of antibiotics in the previous 12 months			
No^b^	1.00		
Yes	1.66	1.08–2.54	0.020
Knowledge about antibiotics and AMR			
Poor^b^	1.00		
Good	0.49	0.32–0.76	0.001
Gender			
Male^b^	1.00		
Female	0.43	0.27–0.70	0.001
Education level			
Secondary school or lower^b^	1.00		
High school	Backward elimination		
Bachelor/University/Doctoral degree	0.56	0.32–0.99	0.048
Marital status			
Others^b^	1.00		
Married	0.71	0.42–1.18	0.186
Employment status			
Unemployed^b^	1.00		
Employed	0.75	0.47–1.20	0.226
Nationality			
Italian^b^	1.00		
Other	1.78	0.69–4.62	0.234
Age, ordinal	0.87	0.70–1.10	0.249
Having interrupted an antibiotic course			
No^b^	1.00		
Yes	1.22	0.78–1.89	0.381
*Model 4. Outcome: Self-medication with antibiotics* ^e^			
*Log-likelihood = −324.59, χ* ^2^ = *50.75, P* < *0.0001, No. of obs.* = *568*			
Having interrupted an antibiotic course			
No^b^	1.00		
Yes	2.14	1.44–3.17	<0.001
Use of antibiotics in the previous 12 months			
No^b^	1.00		
Yes	1.95	1.33–2.86	0.001
Having taken an antibiotic after a phone consultation with the GP			
No^b^	1.00		
Yes	1.72	1.17–2.51	0.005
Gender			
Male^b^	1.00		
Female	0.67	0.44–1.01	0.055
Education level			
Secondary school or lower^b^	1.00		
High school	1.38	0.86–2.21	0.188
Bachelor/University/Doctoral degree	1.34	0.78–2.29	0.280
Age, ordinal	1.10	0.90–1.33	0.349

a The variable *employment status* was eliminated by backward elimination.

b Reference category.

c The variables *gender*, *nationality* and *age* were eliminated by backward elimination.

d The variables *having at least one minor son/daughter who had used antibiotics in the previous 12 months*, *having purchased an antibiotic without a prescription* and *storing leftover antibiotics* were eliminated by backward elimination.

e The variables *employment status*, *marital status*, *having at least one minor son/daughter who had used antibiotics in the previous 12 months* and *knowledge about antibiotics and AMR* were eliminated by backward elimination.

### Practices regarding antibiotics use and self-medication

Table 3 displays reported practices about antibiotic use and self-medication. Of all the subjects who were interviewed, almost half (47.9%) had used antibiotics in the previous 12 months; in particular, 62.1% of users had used them once, 30.9% twice and 7% three or more times. The most reported reasons for using were empirical or targeted therapy of infections (76.2%) and dental procedure prophylaxis (12.2%). More than half (55.4%) reported to have used antibiotics for their minor sons/daughters in the previous 12 months.

**Table 3.** Practices regarding antibiotic use and self-medication

Questions	Yes	
	*N*	%
Have you taken an antibiotic in the previous 12 months? (568)	272	47.9
Has your son/daughter taken an antibiotic in the previous 12 months? (322)^a^	183	55.4
Have you ever taken an antibiotic for the common cold? (568)	64	11.2
Have you ever taken an antibiotic for a fever? (568)	96	16.9
Do you keep leftover antibiotics at home because they might be useful in the future? (568)	386	68
Have you ever used leftover antibiotics without consulting your GP? (386)^b^	92	23.8
Have you ever bought antibiotics without a medical prescription? (568)	145	25.5
Have you ever taken an antibiotic after a phone consultation with the GP, without a clinical examination? (568)	245	43.1
Have you ever interrupted the course of antibiotics prescribed to you [when you started feeling better]? (568)	173	30.5

Numbers of respondents to the statements are in brackets.

a Eligible participants were those with at least a son/daughter ≤17 years.

b Number and percentages referring to the respondents who keep leftover antibiotics at home.

Model 2 showed an independent positive association of having at least one minor son/daughter who had used antibiotics in the previous 12 months (OR = 1.74, 95% CI = 1.19–2.54) with antibiotic use in previous 12 months, whereas an independent negative association with employment status (OR = 0.65, 95% CI = 0.46-0.92) was shown.

When asked about the adherence to the prescribed antibiotic regimen, 30.5% of the study population reported that they had interrupted antibiotic use before they completed the course. The main reasons for stopping taking antibiotics were symptom relief (42.8%), having forgotten to complete the full course of antimicrobial therapy (15.6%) and onset of side effects (34.1%). Furthermore, almost half (43.1%) of the participants stated that they started antibiotic therapy at least once just after a phone consultation with the GP, without a clinical examination (Table 3).

In the sample, 23.6% took antibiotics to treat a common cold and/or fever. The model predicting this practice, with results presented in Table 2, demonstrated sociodemographic, knowledge and other incorrect practices associations. Having taken an antibiotic after a phone consultation with the GP without medical examination (OR = 2.16, 95% CI = 1.41–3.33) and having used antibiotics in the previous 12 months (OR = 1.66, 95% CI = 1.08–2.54) were associated with the incorrect antibiotic use for common cold and/or fever, whereas good knowledge of antibiotics and AMR (OR = 0.49, 95% CI = 0.32–0.76), female gender (OR = 0.43, 95% CI = 0.27–0.70) and having a bachelor/university/doctoral degree (OR = 0.56, 95% CI = 0.32–1.00) compared with those having a secondary school level or lower showed independent positive associations with the responsible practice of not using antibiotics for common cold and/or fever.

A quarter of the sample (25.5%) self-reported to have bought antibiotics without a prescription and 68% to have kept leftover antibiotics at home, and almost a quarter (23.8%) of them reported using antibiotics leftover from a previous course without consulting a GP. Overall, 30.6% of the sample was classified as an antibiotic self-medication user, namely they had taken leftover antibiotics and/or had bought antibiotics without a prescription. Model 4 showed an independent positive association of the interruption of an antibiotic course (OR = 2.14, 95% CI = 1.44–3.17), of the use of antibiotics in the previous 12 months (OR = 1.95, 95% CI = 1.33–2.86) and of the consumption of an antibiotic after a phone consultation with the GP without medical examination (OR = 1.72, 95% CI = 1.17–2.51) with self-medication with antibiotics (Table 2).

## DISCUSSION

This study has tried to investigate the knowledge and practices of the general population regarding antibiotic use and AMR. The findings of the present survey provide an up-to-date insight that will aid in the design of the community educational campaigns to promote prudent antibiotic use.

### Knowledge of antibiotics and AMR

Our results showed fairly poor knowledge of antibiotic use and AMR among the Italian population with less than 2-in-10 that correctly responded to all statements about the investigated knowledge. Many subjects failed to identify that antibiotics have no significant therapeutic effects on viruses. Similarly, European as well as international studies on the public knowledge of antibiotic use indicated a widespread ignorance regarding the ineffectiveness of antibiotic treatment for viral infections [13, 23–25]. The fact that almost one third of the study population believed that infections, regardless of etiology, respond to antibiotics, demonstrates clear misconceptions and confusion regarding the correct indication for antibiotic use. Although it is not a so vast proportion of the study population, it deserves attention. Indeed, it could lead to the patient demand for antibiotics for viral infections, such as acute respiratory tract infections (RTIs) that are frequent reasons for medical consultation in general practice and are commonly caused by viruses and do not require antibiotics. Indeed a very high frequency of nonevidence-based prescription of antibiotics at primary care among the adult population with RTIs was reported in the same area [26].

Confusion concerning the phenomenon of AMR was also detected. Among knowledge statements, those regarding AMR showed the highest percentage of failure, and these findings pointed out that the recognition of AMR and the measures for its prevention remain partially unknown among the public in Italy. The results of multivariate analysis underlined the heterogeneity of public knowledge regarding antibiotics and AMR on the basis of sociodemographic factors. These figures could be helpful as a considerable inter- and intraregional variability in antibiotic consumption and prescribing pattern has been already described in Italy [27] as well as its relationship with sociodemographic factors [28]. Our findings indicated that males, foreign nationals and those with a lower level of education had poor knowledge of antibiotics and AMR and could be highly useful in planning tailored educational interventions.

### Practices regarding antibiotic use and self-medication

It is worthwhile to underline that the proportion of individuals who reported having taken at least one antibiotic for himself/herself (47.9%) or for their minor son/daughter (55.4%) in the previous 12 months is among the highest reported in European Member States, emphasizing that antibiotic consumption is considerable in the study area as well as in the country as a whole [29]. A similar antibiotic consumption prevalence (46.8%) was observed in a retrospective analysis of reimbursement pharmacy records in the outpatient setting of another Region of Southern Italy [28].

We investigated two types of practices that are known to contribute to self-medication: obtaining antibiotics from a pharmacy without a prescription and using leftover antibiotics from a previous course. In Italy, availability of over-the-counter antibiotics without prescription has already been described [4], although outpatient antimicrobials are law restricted to prescription-only use, similar to most European countries [15]. The study findings underscore that one third of the sample had been involved in self-medication with antibiotics. This is of concern because the world literature indicates that self-medication plays a crucial role in driving resistance [15, 30, 31]. Policy makers should pay special attention to the enforcement of laws prohibiting the sales of antibiotics without prescription and to the regulations of leftover antibiotic use aimed at dispensing precise doses instead of whole packages of antibiotics. Indeed, in Italy, antibiotics are dispensed in fixed packs instead of in an exact number of tablets and no measures to counteract the use of leftover antibiotics, such as take-back programs, including the return of unused or excess drugs to pharmacies, are promoted.

The findings of multivariate analysis identified groups of participants who were more prone to use self-medication antibiotics as well as to take antibiotics without an indication (a common cold and/or fever). As expected, respondents’ adequate knowledge of antibiotics was identified to positively correlate with correct antibiotic use, which is consistent with previous studies [10, 23, 32]. This finding emphasizes that public health professionals have to better evaluate the state of public knowledge as a precursor to the drawing of effective educational campaigns, as previously suggested [33]. Indeed, a previous European study demonstrates that targeted media campaigns at those with low knowledge are likely to change their usage habits [13]. Moreover, the consumption of an antibiotic after a phone consultation with the GP without medical examination was another potential predictor of wrong practices. Although this practice could not necessarily affect the appropriateness of prescription, the proportion of the study participants (43.1%) that started antibiotic therapy just after a phone consultation with the GP is quite high, especially in Italy, where one of the highest rates of antibiotic consumption in Europe has been reported, and prudent use of antibiotics should be broadly encouraged. In the local context, access to telemedicine visits in which patients are connected to physicians outside of the medical ambulatory is not a well-established procedure, and the same standards of in-person care cannot be assured. Evidence from a previous retrospective cohort study comparing the quality of antibiotic prescribing for acute RTIs among children across three settings, demonstrated that receiving a guideline-concordant antibiotic prescription was less likely at telemedicine visits compared with matched visits at other clinical settings (i.e. urgent care, GP) [34].

The current finding that almost 3-in-10 of those being prescribed an antibiotic did not follow the established regimen and discontinue treatment prematurely mainly due to relief of symptoms is of concern. Similar results were obtained in studies conducted in other countries, including the UK (11.3%) [35], Portugal (55.7%) [36], Croatia (29.9%) [37] and Romania (31.9%) [23]. This misconception in antibiotic use may put the patient at risk of relapse with multidrug-resistant bacteria. It is widely recognized that inadequate dosing and incomplete courses have contributed to the emergence and spread of antibiotic resistance [31].

In our study, the mass media, including the Internet, were indicated as the main sources of AMR information. The role of the Internet as a common source of health- and antibiotic-related information was reported by previous research conducted in the Italian population [38, 39]. Health organizations must consider the Web within their communication strategies to promote the appropriate use of antibiotics. It is worth mentioning that only 2 out of 10 participants mentioned HCWs, whereas they could play a key role in changing public views and encouraging prudent use of antibiotics [15]. Moreover, the finding that a quarter of the sample has bought antibiotics without a prescription suggests that this has become a worrisome practice in Italy. In addition to using leftover antibiotics, the main cause for nonprescription antibiotics is over-the-counter selling in pharmacies. This incorrect practice highlights that pharmacists are an additional key element that should be the target of specific interventions, and that studies aimed at clarifying the potential drivers of dispensing of antibiotics without prescription are needed.

### Strengths and limitations

The strengths of this study include a high response rate, an even distribution of men and women, all the desired age groups and subjects having high as well as low levels of education. The high response rate of a survey is a key data quality indicator since reducing nonresponse bias coupled with random sampling ensures generalizability of findings to the population of interest. Another strength is that the respondents did not have the opportunity to take the questionnaires home. Consequently, they could not look up the correct answers online or confer with someone else before responding, that could lead to some overestimation of knowledge and of correct practices regarding antibiotic use. Limitations of this study attain to the cross-sectional design, not allowing to draw conclusions on causality about the observed associations, and to the self-reporting of practices, only a proxy of real practices. Another of the study’s limitation is the potential overestimation of positive outcomes. It is possible that people who were more informed or interested in the topic were more willing to participate, but the high response rate reduces this limitation to a minimum. To overcome the difficulty of identifying a sampling frame of all primary care patients, we first selected a random sample of clusters (27 clinics), and then all consecutive subjects attending GP and CBP clinics during randomly selected days were invited to participate. Since enrollment of patients within practices was not random, issues of representativeness may arise and indeed selected patients may be representative of those who attend GP and CBP clinics. However, in an attempt to overcome this limit, we compared the sociodemographic characteristics of the study sample in some clinics with those of all the patients referring to that specific clinic, and no substantial differences were found. Furthermore, our study involved two Italian Regions, which might not represent all the adult population in Italy. However, we are confident that the findings of the study may be representative at least for the Regions of Southern Italy.

## CONCLUSIONS AND IMPLICATION

Even with these potential limitations, the findings of the present study highlighted a considerable antibiotic consumption in the adult population of Southern Italy together with misconceptions regarding the correct indication for antibiotic use that could foster indiscriminate antibiotic use. Policy makers should consider multifaceted interventions to tackle the determinants of antibiotic misuse at the healthcare system as well as at the patient level.

## FUNDING

No external funding for this manuscript.

**Conflicts of interest:** None declared.
